# Intervention Strategies Used in Sport Injury Prevention Studies: A Systematic Review Identifying Studies Applying the Haddon Matrix

**DOI:** 10.1007/s40279-017-0718-y

**Published:** 2017-03-16

**Authors:** Ingrid Vriend, Vincent Gouttebarge, Caroline F. Finch, Willem van Mechelen, Evert A. L. M. Verhagen

**Affiliations:** 1 Department of Public and Occupational Health and Amsterdam Public Health Research Institute, VU University Medical Center, Van der Boechorststraat 7, 1081 BT Amsterdam, The Netherlands; 2 Amsterdam Collaboration on Health and Safety in Sports, IOC Research Center, AMC/VUmc, Amsterdam, The Netherlands; 3 Consumer Safety Institute VeiligheidNL, Amsterdam, The Netherlands; 4 School of Human Movement and Nutrition Sciences, Faculty of Health and Behavioural Sciences, University of Queensland, Brisbane, Australia; 5 Division of Exercise Science and Sports Medicine (ESSM), Department of Human Biology, Faculty of Health Sciences, University of Cape Town, Cape Town, South Africa; 6 Australian Collaboration for Research into Injury in Sport and its Prevention (ACRISP), Federation University Australia, Ballarat, Australia

## Abstract

**Background:**

Prevention of sport injuries is crucial to maximise the health and societal benefits of a physically active lifestyle. To strengthen the translation and implementation of the available evidence base on effective preventive measures, a range of potentially relevant strategies should be considered.

**Objective:**

Our aim was to identify and categorise intervention strategies for the prevention of acute sport injuries evaluated in the scientific literature, applying the Haddon matrix, and identify potential knowledge gaps.

**Methods:**

Five electronic databases were searched (PubMed, EMBASE, SPORTDiscus, CINAHL, Cochrane) for studies that evaluated the effect of interventions on the occurrence of acute sport injuries. Studies were required to include a control group/condition, prospective data collection, and a quantitative injury outcome measure.

**Results:**

A total of 155 studies were included, mostly randomised controlled trials (43%). The majority of studies (55%) focussed on strategies requiring a behavioural change on the part of athletes. Studies predominantly evaluated the preventive effect of various training programmes targeted at the ‘pre-event’ phase (*n* = 73) and the use of equipment to avoid injury in the ‘event phase’ (*n* = 29). A limited number of studies evaluated the preventive effect of strategies geared at rules and regulations (*n* = 14), and contextual modifications (*n* = 18). Studies specifically aimed at preventing re-injuries were a minority (*n* = 8), and were mostly related to ankle sprains (*n* = 5).

**Conclusions:**

Valuable insight into the extent of the evidence base of sport injury prevention studies was obtained for 20 potential intervention strategies. This approach can be used to monitor potential gaps in the knowledge base on sport injury prevention.

## Key Points

**Table:** 

A modified version of the Haddon matrix, representing 20 possible intervention strategies, is a useful tool to identify possible intervention strategies for sport injury prevention.
Studies in the area of rule and regulation changes, education, and psychological/cognitive skills training are underrepresented. These provide new opportunities for sport injury prevention research.
Non(randomised) controlled trials have been used extensively in sport injury prevention studies, and are valid options to evaluate the effect of intervention strategies when the use of a control group is not feasible, for instance, in the case of rule modifications and policy interventions.

## Introduction

Both a physically active lifestyle and sport participation are recommended because of their inherent health benefits [1–4]. However, they also carry a risk of sustaining injuries. These injuries form a significant public health problem at an individual and societal level, including (temporary) physical inactivity and direct and indirect costs related to medical treatment and work absenteeism. As such, the prevention of sport injuries is important to maintain and increase a physically active lifestyle and sports participation, and to maximise the related health and societal benefits [5].

Numerous studies and systematic reviews have evaluated the effects of preventive interventions on the risk of sport injuries [6–12], and, as such, these provide an evidence base for implementation efforts [13]. Differences have been found in the type of preventive measure or intervention under study by injury type and sport [8–10]. Most studies have used a randomised controlled trial (RCT) design [11]. RCTs are considered the optimal study design to establish a cause–effect relationship and, as such, to establish the effect of an intervention [14–16]. Other study designs have also been used in sport injury prevention studies [11], as RCTs are not always feasible in a real-world sport setting due to ethical or practical reasons [14, 15]. This is especially true for evaluating contextual, policy-level interventions (such as legislation or regulation changes) and for interventions that have become common practice. When evaluating such interventions, time trend analyses (e.g. pretest–posttest designs) are considered adequate study designs [14, 15].

Despite this wide base of knowledge on sport injury prevention, large-scale implementation of effective preventive interventions in real-world sport settings is still a major challenge [17–19]. Actual injury prevention in daily practice requires large-scale adoption and the correct use of evidence-based preventive interventions by the target population [13]. The majority of the available evidence on sport injury prevention appears to focus on the behaviours and actions of individual athletes, including evaluating the use of personal protective equipment (PPE) and specific training programmes to reduce the risk of injuries [7, 11, 20]. Implementation of such measures requires a behavioural change on the part of an athlete [21, 22]. This may be a challenging task, since intervention strategies that predominantly target behavioural modifications in individuals are found to be less effective in injury prevention than those based on contextual modifications, such as regulations, enforcement methods, and environmental and product modifications [7, 22–24]. Moreover, in the sport injury context, injury prevention requires more than just a change in athlete behaviours, but also relies on broad support and behaviour change from sporting federations, coaches, allied health staff and others [25]. Therefore, a range of potentially relevant strategies should be considered to support and strengthen sport injury prevention efforts.

An overview of sport injury prevention studies categorised by their intervention strategy, i.e. geared at the individual versus geared at the context, is as yet lacking. A useful and valid tool for the categorisation of intervention strategies for the prevention of acute injuries is the Haddon matrix [24, 26]. This matrix, originating from traffic safety research, has previously been successfully applied to sport injury prevention. An early example of its use to identify possible sport injury prevention strategies is the study by Bahr et al. [27] for the prevention of ankle sprains in volleyball. A recent review on snow sport injuries also used the Haddon matrix as its conceptual framework [28].

The aim of this systematic review was to identify intervention strategies for the prevention of sport injuries evaluated in the scientific literature, and to identify potential intervention strategies not yet evaluated (i.e. to identify potential knowledge gaps), making use of the Haddon matrix. The review was restricted to the prevention of acute sport injuries. The specific objectives of this review were to (1) provide a categorisation of sport injury prevention studies by intervention strategy using the Haddon matrix; (2) assess differences in intervention strategies evaluated in studies aimed at the prevention of different injury types and sports; and (3) categorise the number of sport injury prevention studies by study design and intervention strategy. Such an evidence-based overview can facilitate future sport injury prevention efforts by identifying possible strategies to choose from, given an injury problem and context.

## Methods

### Definitions

For the purpose of this review, sport injury prevention studies were defined as studies evaluating the efficacy or effectiveness of interventions aiming to prevent the occurrence of injuries within a real-world sport setting [25]. Acute sport injuries were defined as traumatic injuries (i.e. caused by a single, specific and identifiable onset), in contrast to overuse injuries (i.e. a gradual onset) [29] and systemic injuries (e.g. heat stress, organ failure, sudden cardiovascular death).

### Literature Search

A systematic computerised search was performed to identify relevant studies published up to 31 December 2015, using five electronic databases: PubMed, EMBASE, SPORTDiscus, CINAHL and Cochrane Central Register of Controlled Trials. The search terms used were a combination of database-specific thesaurus terms and free-text terms in the title and abstract related to (a) the problem (injur* AND sport*/athlet*/exercis*), (b) the intervention (prevent* AND injur*), and (c) the study design, using standard Cochrane scripts (terms were used to identify clinical trials, cohort, epidemiological and evaluation studies, and systematic reviews). The search was limited to humans and English-language publications. The reference lists of relevant recent systematic reviews (i.e. published since 2010) that appeared in the search were screened for additional studies. No publication date restrictions were used.

#### Inclusion Criteria

Studies were considered for inclusion if they met all of the following criteria: (a) they evaluated the effect of a preventive measure or intervention on the occurrence of acute injuries in sports; (b) the study subjects were able-bodied, healthy and physically active at the time of injury (all ages, male and female); (c) data were registered prospectively; (d) the study design included a control group or control condition (e.g. pre-interrupted data serving as control condition in pretest–posttest design, or interrupted time series); (e) the study results contained a quantitative injury measure as an outcome; and (f) the article concerned original research, published in a peer-reviewed journal.

#### Exclusion Criteria

Studies that evaluated the effect of a preventive measure or intervention on overuse injuries were excluded. However, studies targeting both acute and overuse injuries [or all injuries in specific body region(s)] were included in the review, but data extraction was restricted to acute injuries only. Injury prevention studies related to commuting (e.g. cycling), dance, performing arts (e.g. ballet and circus), and leisure time physical activity next to sports (e.g. play) [30] were excluded from this review. Injury prevention studies evaluating the effect of interventions outside an everyday sport setting (i.e. military training studies, laboratory-based studies, and modelling studies) were excluded. Studies that reported on intermediary behaviour (e.g. protective equipment use) or determinants of preventive behaviour (e.g. individuals’ knowledge or attitudes) as an outcome measure, rather than reporting on a quantitative injury measure as an outcome, were not included either. If several exclusion criteria applied to a study, only one was noted.

#### Study Selection

All identified studies were screened for relevance in two steps. First, all studies were evaluated for inclusion based on title and abstract. In the case of uncertainty, full-text articles were retrieved. To become familiarised with the inclusion assessment, two reviewers (IV and EALMV) independently screened a random selection of 215 studies in two rounds. Out of the first 106 studies screened, there was initial disagreement on 16 studies; the next 109 studies screened resulted in disagreement on one study. Based on this high level of agreement, it was decided that the remaining studies only needed to be evaluated for inclusion based on title and abstract by one reviewer (IV). As a second step, two authors independently evaluated full-text articles for final inclusion (IV and EALMV). Any disagreement in the selection of potentially relevant studies was resolved by consensus.

### Methodological Quality Assessment

All relevant studies were categorised by study design following the system used in evidence-based practice to indicate the strength of evidence based on the study results [31]. As the primary aim of this systematic review was to categorise studies by intervention strategy used, and not to assess the effect of a preventive intervention, risk of bias in individual studies was not assessed. A similar approach has been used in previous systematic reviews on the prevention of sports injuries [7, 11].

### Data Extraction

One reviewer (IV) extracted data from the included studies, describing study design, characteristics of study participants, sport, injury (causation, location and type), preventive intervention, study outcome, and intervention strategy (Table 1). A standardised form was used for data extraction. The primary aim of each individual sport injury prevention study was used as a starting point for the categorisation of the extracted data. The categorisation of extracted data was checked for consistency.

**Table 1:** Data extracted from the included studies

Item	Categories
Study design	Randomised controlled trial; controlled trial; prospective cohort study; pretest–posttest design; interrupted time series
Target population	General sport population; athletes with a previous injury (or reduced function/residual symptoms)
Age	Children (<18 years); adults (18–65 years); elderly (65+ years); all
Sex	Male; female; both
Sport	Sport activity targeted in the intervention under study
Preventive intervention	Training (strength, plyometrics, endurance, agility, flexibility, stretching, balance/coordination, sport-specific skills/technique, other); education; rules and regulations (rule change, enforcement); equipment (personal protective equipment, brace, tape, footwear/orthotics, sport devices); context (physical, sociocultural, policy); multi-component intervention
Intervention target^a^	Athlete; rules and regulations; equipment; sport setting or context; multiple
Time window^a^	Pre-event; event; post-event; multiple
Injury causation^b^	Acute (traumatic onset); overuse (gradual onset)
Injury location (body region)^b^	Head/face, neck/cervical spine (head/neck); shoulder/clavicle, arm/elbow, wrist, hand/fingers (upper limb); back, abdomen, pelvis (trunk); groin, thigh/hamstring, knee, lower leg/Achilles tendon, ankle, foot/toes (lower limb); other
Injury type (structure involved)^b^	Fracture (bone); dislocation/subluxation, sprain (joint-ligament); strain, tendinopathy (muscle–tendon); abrasion, laceration, contusion (skin); concussion, structural brain injury, spinal cord injury (central and peripheral nervous systems); dental injury; organ injury (blunt trauma); other
Study outcome	Significant change; not significant change in main injury outcome(s) (following the study outcome and the level of statistical significance set by the original researchers)

^a^Adapted from the Haddon matrix [24, 26]

^b^Based on the Orchard Sports Injury Classification System (OSICS): sprain = stretch and/or tear of a ligament; strain = stretch and/or tear of a musculotendinous structure) [29]

#### Intervention Strategy

The included studies were categorised by their intervention strategy, applying a modified version of the original Haddon matrix. The original Haddon matrix identified nine potential intervention strategies to prevent injuries, based on two dimensions (3 × 3 matrix): (1) three levels for intervention targets (i.e. 1 = host, 2 = agent, 3 = physical and sociocultural environment) and (2) the time window or time frame in which an injury occurs (i.e. 1 = pre-event, 2 = event, 3 = post-event) [24, 26].

For the purpose of this review, the original Haddon matrix was modified for sport injury prevention. The first dimension (i.e. intervention target) was expanded from three to four levels. The host was interpreted as the athlete; the agent as the sport activity subdivided into rules and regulations of the sport, and sport equipment; and the environment was interpreted as the physical, sociocultural and policy setting or context within which the sports injury occurs [24, 27]. Interventions targeting the agent are aimed at reducing the amount of energy created or transferred. The second dimension (i.e. time window) comprised the three levels of the original Haddon matrix. In accordance with the purpose of this review, the post-event phase was restricted to interventions specifically targeted at the prevention of recurrent injuries. Next, a category was added to both dimensions of the original Haddon matrix to categorise studies evaluating the effect of multi-component or multiple interventions. As such, a total of 20 potential intervention strategies for sport injury prevention were distinguished, based on two dimensions (5 × 4 matrix; Table 2).

**Table 2:** Definitions used for the modified Haddon matrix with regard to the prevention of acute sport injuries [24, 27]

Dimension level	Definition
Dimension A: intervention target	
Athlete (*host*)	Interventions targeted to change individual player attitudes, knowledge or behaviours (e.g. improve physical fitness, skills and techniques)
Rules and regulations in sport (*agent*)	New or modified rules in sport (including rules regulating PPE use, and enforcement of rules) to change athletes’ behaviour related to the sport activity
Sport equipment (*agent*)	New or modified PPE or sport equipment related to the sport activity (including tape, braces, footwear and shoe inserts)
Sport setting or context (*environment*)	Interventions targeted to change the physical, sociocultural and policy setting or context within which the sport injury occurs
Multi-component, or multiple interventions	Interventions that include multiple intervention targets
Dimension B: time window or time frame in which an injury occurs	
Pre-event	Interventions aimed to prevent the sport injury event from occurring in the first place, reduce the injury risk to an acceptable level before participation, or build the capacity of an athlete before the injury event
Event	Interventions aimed at being effective at the time of the injury event
Post-event	Interventions aimed to minimise the consequences of a sports injury by treatment and rehabilitation, and returning the athlete to the ‘pre-event’ status
Multiple time windows	Interventions that include multiple interventions, targeting different time windows in which an injury occurs (within a study)

*PPE* personal protective equipment

## Results

### Literature Search

**Fig. 1:**
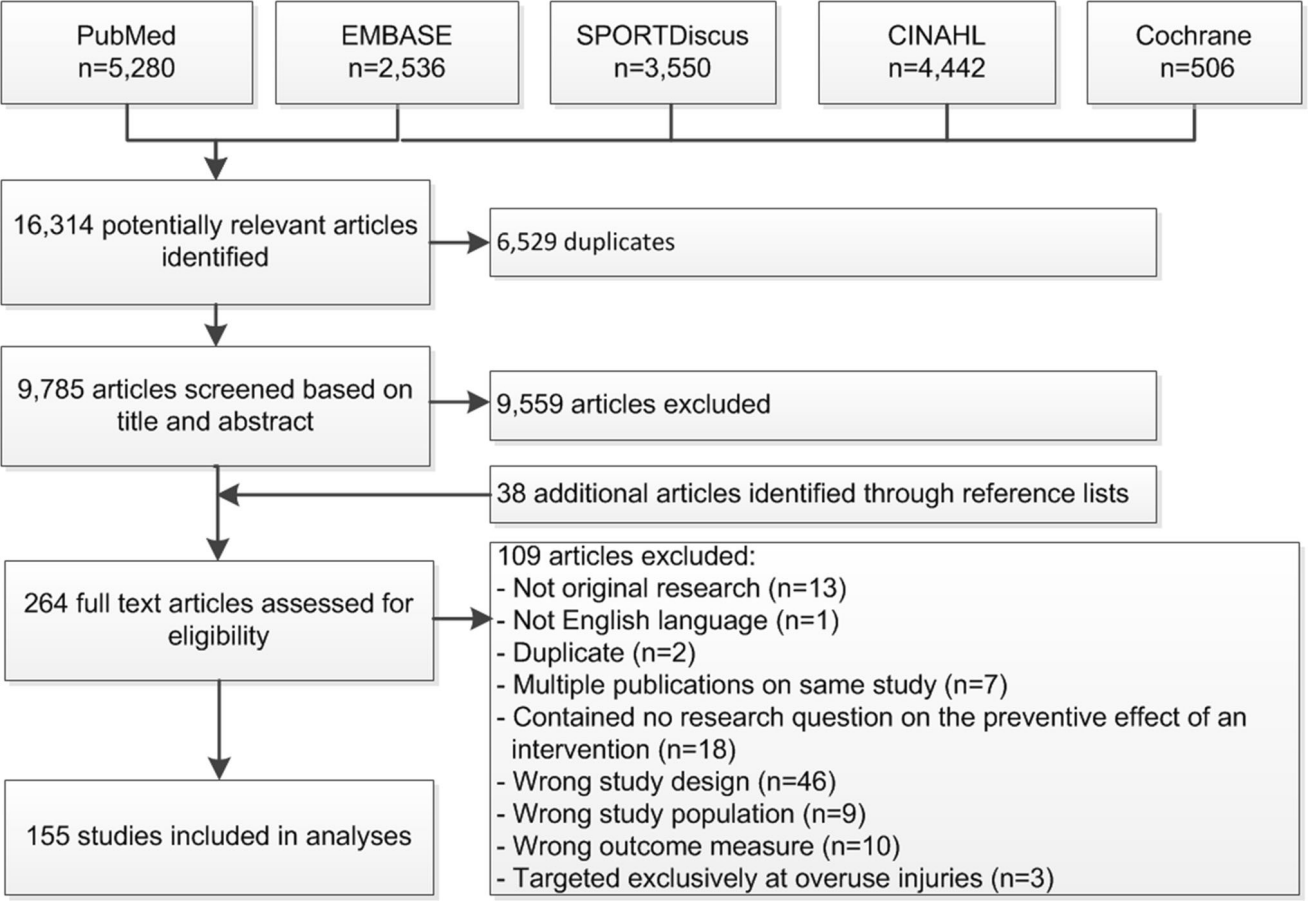
Flow chart of literature search and study selection. *CINAHL* Cumulative Index to Nursing and Allied Health Literature, *Cochrane* Cochrane Central Register of Controlled Trials

The search strategy initially yielded 16,314 articles, of which 226 studies were considered relevant after title and abstract screening. An additional 38 articles were identified through reference lists of relevant systematic reviews. After reading the full-text papers, a further 109 papers were excluded, including three studies exclusively targeted at the prevention of overuse injuries [32–34]. A total of 155 studies were included for analyses (Fig. 1). Of these, 88 studies targeted the prevention of acute injuries and 67 studies the prevention of all injuries, including acute injuries. Most studies used an RCT design to evaluate the preventive effect of an intervention (*n* = 66; 43%) [30, 35–99]. In addition, 23 controlled trials (CTs) (15%) [100–122], 22 prospective cohort studies (14%) [123–144], 39 studies with pretest–posttest designs (25%) [145–183], and five interrupted time series (3%) [184–188] were included.

#### Target Study Population

The majority of the included studies focussed on preventing injuries in the general sport population regardless of injury history (*n* = 135). Some studies exclusively targeted the prevention of re-injuries (i.e. athletes with a previous injury or reduced function/residual complaints; *n* = 8) [40, 47, 52, 56, 59, 69, 99, 140], or included athletes at risk based on a psychological high injury risk profile [93, 94] or reduced hip adductor strength [174] (*n* = 3). Another nine studies excluded athletes with a previous (recent) injury at the start of the study [36, 38, 39, 46, 75, 88, 105, 123, 131].

A total of 25 different sports were studied. Soccer was the most frequently studied sport (*n* = 43; 28%), followed by rugby (*n* = 13; 8%), American Football (*n* = 12; 8%), basketball (*n* = 11; 7%), and ice hockey (*n* = 10; 7%). Another 13 studies (8%) focussed on the prevention of injuries in multiple sports combined.

One-third of the included studies were targeted at male athletes only (*n* = 52; 34%). Another 22 studies only included females (14%), and 49 studies (32%) included both sexes. The focus of the included studies was on the prevention of sport injuries in children (*n* = 49; 32%), adults (*n* = 40; 26%), or people of any age (*n* = 34; 22%). For 18 studies (12%), the age of the study population could not be retrieved.

#### Body Region and Injury Type

Overall, most studies evaluated the effect of an intervention on injuries to the lower limb (*n* = 73), and/or any injury (*n* = 72). With regard to the lower limb, the majority of studies specifically targeted the prevention of ankle injuries (*n* = 27) and/or knee injuries (*n* = 23). There were 24 studies specifically aimed at preventing ankle sprains, and 13 studies aimed at preventing knee ligament injuries. A total of 25 studies aimed to prevent head/neck injuries, primarily head/face injuries (*n* = 21) including concussions (*n* = 10). A few studies specifically targeted sport injuries to the upper limb (*n* = 4) and/or trunk (*n* = 6).

### Intervention Strategies

#### Preventive Interventions Under Study

Most studies (*n* = 70; 45%) focussed on the preventive effect of a variety of training programmes, including warm-up programmes and the FIFA 11/11+ programme, aimed at improving general physical fitness and/or skills of athletes. The focus of 33 studies (21%) was on the preventive effect of sport equipment, including PPE, and brace or tape. Another 14 studies evaluated the preventive effect of rules and regulations in sport (9%), including rule modifications (*n* = 4), stricter rule enforcement by referees (*n* = 2), and (new/existing) rules related to mandatory PPE use (*n* = 8). The effect of education was evaluated in 12 studies (8%), other context-related interventions in 12 studies (8%), and multi-component interventions/multiple interventions in 14 studies (9%).

#### Strategies Used in Sport Injury Prevention Studies

The majority of intervention strategies targeted the preventive behaviour of athletes in the pre-event phase (*n* = 79; 51%). These strategies most often concerned training programmes to improve physical fitness (*n* = 58); training components frequently included were strength training (58%), balance/coordination training (45%), stretching (31%), and plyometrics (30%). Another six training programmes (9%) in the pre-event phase were aimed at improving psychological and/or cognitive skills [86, 93, 94, 106, 121, 122].

A total of 29 studies evaluated the effect of sport equipment use (i.e. PPE, tape, brace and footwear) in the event phase (19%). Few injury prevention studies were found on the effect of strategies targeted at rules and regulations (*n* = 14) or contextual modifications (*n* = 18). These strategies were primarily implemented at an (inter)national level (71% and 61%, respectively). Very few studies targeted the use of sport equipment in the pre-event phase (*n* = 2), athletes in the event phase (one study on teaching falling, landing and recovery skills in Australian Football players) [118], or strategies in the post-event phase (*n* = 8). Interventions in the post-event phase primarily aimed to prevent recurrent ankle sprains (*n* = 5), with the main focus on training programmes (Table 3).

**Table 3:** Studies of the prevention of acute sport injuries, categorised by preventive intervention and intervention strategy following the modified Haddon matrix (*n* = 155 studies)

Intervention target	Time window			
	Pre-event (*n* = 98; 63%)	Event (*n* = 44; 28%)	Post-event (*n* = 8; 5%)	Multiple time windows (*n* = 5; 3%)
Athlete (*n* = 85; 55%)	Training programme to improve^a^: – Physical fitness (*n* = 49)^b^ – FIFA 11/11 + (*n* = 9) [53, 64, 72, 76, 81, 90, 91, 107, 156] – Psychological/cognitive skills (*n* = 6) [86, 93, 94, 106, 121, 122] Education (*n* = 6): – Increase risk awareness (*n* = 2) [35, 180] – Varied information on injury preventive behaviour (*n* = 4) [41, 61, 110, 175] Multi-component (*n* = 9) [30, 43, 108, 111, 147, 153, 157, 162, 168]	Training programme to improve falling, landing and recovery skills (*n* = 1) [118]	Balance training (*n* = 4) [40, 56, 59, 99] Balance and strength training (*n* = 1) [47]	None
Rules and regulations (sport activity) (*n* = 14; 9%)	New or modified rules of sport (*n* = 2) [154, 166] New law (*n* = 1) [145] Strict enforcement of rules/penalising (*n* = 2) [148, 151]	Mandatory use of PPE (*n* = 8) [125, 133, 134, 169, 172, 183, 184, 187]	None	Modify the rules of sport (*n* = 1) [188]
Equipment (sport activity) (*n* = 33; 21%)	Introduction of carving skis (*n* = 1) [150] Use of (appropriate) footwear (*n* = 1) [62]	Use of (appropriate): – PPE (*n* = 14) [48, 57, 65, 68, 75, 83, 87, 104, 114, 120, 124, 141, 143, 177] – Tape (*n* = 2) [50, 115] – Brace (*n* = 9) [58, 67, 74, 95, 97, 123, 132, 138, 139] – Footwear/orthotics (*n* = 2) [37, 131] – Multiple (tape, brace, PPE) (*n* = 2) [137, 142]	Use of thermal pants (*n* = 1) [140]	Use of braces (*n* = 1) [82]
Context (environment) (*n* = 18; 12%)	Coaching education (*n* = 4) [149, 155, 163, 186] Referee education (*n* = 1) [96] Changing safety culture (fair play programme) (*n* = 1) [130] Policy change^c^ (*n* = 5) [135, 158, 159, 161, 185]	Policy for mandatory use of: – Braces by coaches (*n* = 2) [127, 171] – PPE/sport equipment by league (*n* = 1) [136] – PPE by school (*n* = 1) [178] Use of breakaway bases in softball (*n* = 1) [109]	Rehabilitation programme, including return to play criteria (*n* = 1) [52]	Coaching education/concussion side-line management tool (*n* = 1) [182]
Multi-component/multiple interventions (*n* = 5; 3%)	Education (fair play), and new policy on cancelling games (*n* = 1) [160]	Rule change, use of (appropriate) PPE and brace (*n* = 1) [164]	Training programme (balance/strength)/use of braces (*n* = 1) [69]	Training programme (balance/strength)/use of braces (*n* = 1) [77] Education/fair play/mandatory PPE use/supervision (*n* = 1) [119]

*PPE* personal protective equipment

^a^Including warming-up programmes

^b^[36, 38, 39, 42, 44–46, 49, 51, 54, 55, 60, 63, 66, 70, 71, 73, 78–80, 84, 85, 88, 89, 92, 98, 100–103, 105, 112, 113, 116, 117, 126, 128, 129, 144, 146, 152, 165, 167, 170, 173, 174, 176, 179, 181]

^c^Allowing body checking in ice hockey at a younger age

#### Study Outcome

The majority of the interventions under study focussed on changing the behaviour and actions of individual athletes to reduce the risk of injuries, such as specific training programmes in the pre-event phase, and the use of protective equipment (i.e. PPE, brace and tape) in the event phase (Table 4). Based on the study outcomes reported in the original studies, the evidence base for these intervention strategies was relatively low, with 25–75% of the studies reporting a statistically significant change in injury risk. In contrast, the evidence base for strategies less often studied (e.g. changes of rules and regulations in sport, post-event strategies) was relatively high, with 75% or more studies reporting a significant effect (Table 4).

**Table 4:**
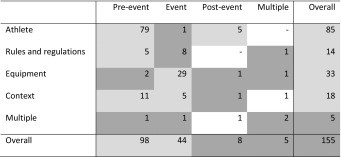
Absolute number of studies reporting the prevention of acute sport injuries categorised by intervention strategy following the modified Haddon matrix, and the proportion of studies with a statistically significant effect

Colour coding indicates the proportion of studies with a statistically significant effect: white <25%; grey 25–75%; dark grey ≥75%

– no studies

### Study Design

**Fig. 2:**
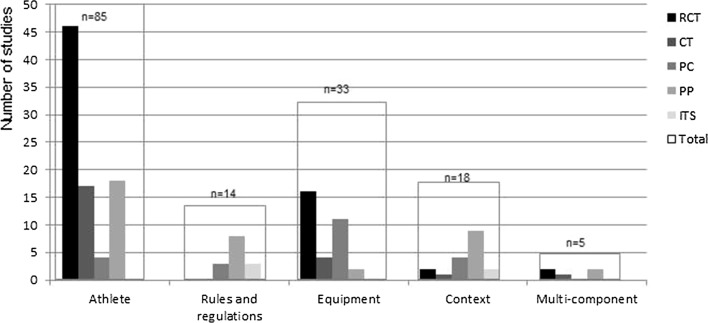
Absolute number of studies categorised by Haddon’s intervention target and study design. *RCT* randomised controlled trial, *CT* controlled trial, *PC* prospective cohort study, *PP* pretest–posttest design, *ITS* interrupted time series

Differences in study design used in individual studies were distinctive when categorised by Haddon’s intervention target (Fig. 2). RCTs (70%) and CTs (74%) were most often used to evaluate the effect of interventions targeted at the athlete. Non-randomised prospective cohort studies were used mostly to evaluate the preventive effect of sport equipment (50%); pretest–posttest designs were used mostly to evaluate the effect of strategies targeted at the athlete (46%), contextual modifications (23%), and rules and regulations in sport (21%).

### Intervention Strategies for Specific Injury Targets

**Fig. 3:**
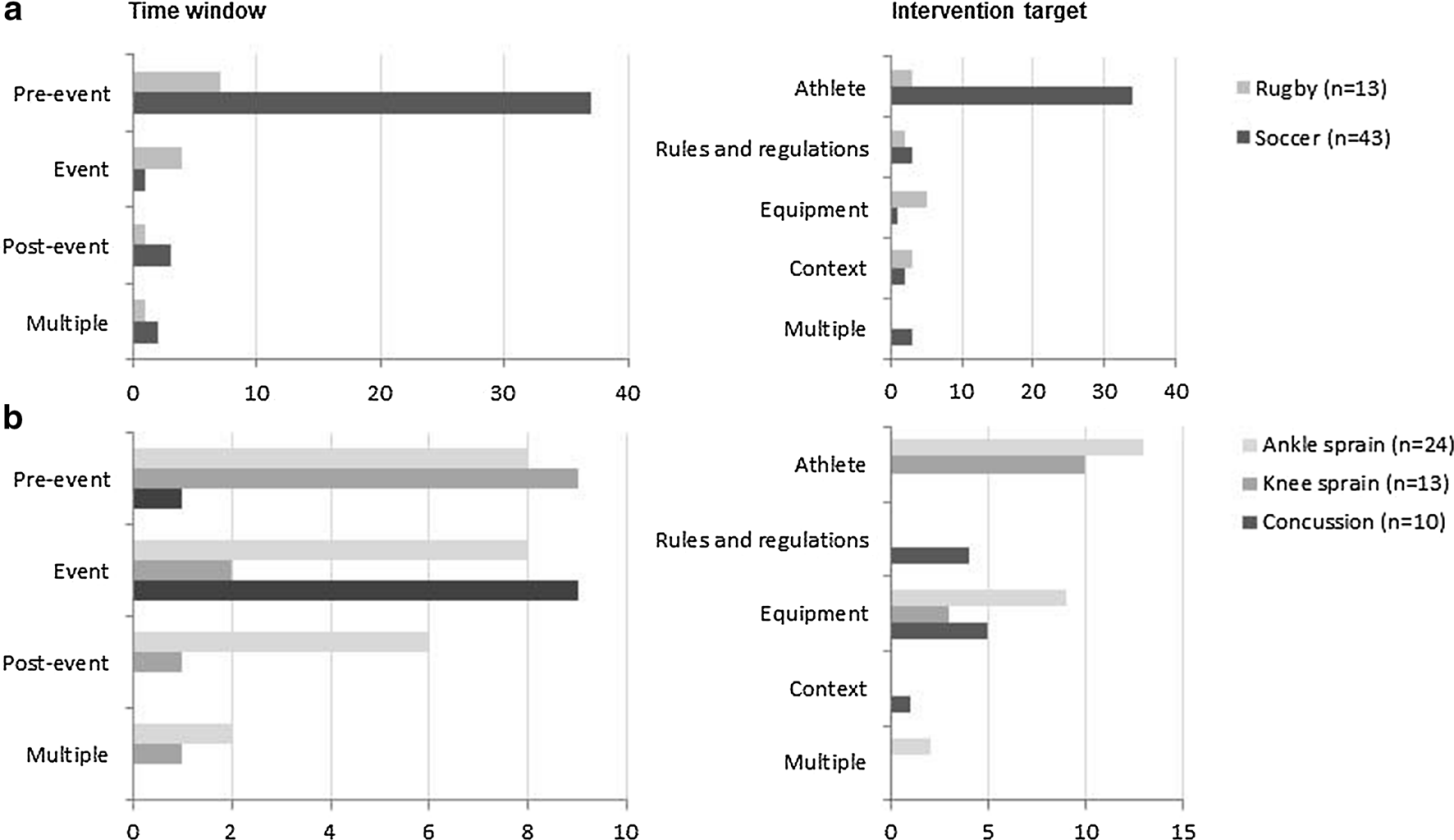
Absolute number of studies targeted at the prevention of soccer or rugby injuries (**a**), and ankle sprains, knee sprains or concussions (**b**), categorised by intervention strategy used

Soccer and rugby were the sports most often targeted in the studies included. In contrast to rugby, the emphasis of prevention studies in soccer was on changing athletes’ behaviour in the pre-event phase (Fig. 3), mostly through training programmes (two and 29 studies, respectively).

Pre-event phase studies frequently focussed on the prevention of ankle (*n* = 8) and knee sprains (*n* = 9), whereas event phase studies had relatively few focussing on the prevention of knee sprains (*n* = 2; Fig. 3). In the event phase, both studies of knee sprain prevention concerned knee bracing [67, 123]; the studies of ankle sprain prevention (*n* = 8) targeted the effect of braces [58, 67, 95, 138], tape [115], shoe design [37, 131], or a combination of these interventions [50]. No studies were found on the effect of changes of rules and regulations or contextual modification to prevent ankle or knee sprains. No evidence was available on the effect of changing athletes’ behaviour (e.g. through education) on the occurrence of concussions in sports. The focus was on the effect of (mandatory) PPE use (*n* = 9; Fig. 3).

## Discussion

The primary aim of this review was to categorise sport injury prevention studies by their intervention strategy, using a modified version of the Haddon matrix. The majority of the available evidence focussed on strategies that required a behavioural change on the part of individual athletes. These studies predominantly evaluated the preventive effect of various training programmes targeted at improving athletes’ level of physical fitness and/or sport-specific skills before the injury event, and the use of PPE, tape or brace aimed at being effective at the time of the injury event. This corresponds to reports in previous reviews of sport injury prevention [11].

The current review showed that research related to some specific intervention strategies is underrepresented. Only a few studies were identified that evaluated the preventive effect of strategies geared at rules and regulations in sport, contextual modifications, and sport equipment (other than PPE, tape or brace) on the occurrence of sport injuries. The lack of studies of the preventive effect of rule modifications to prevent sport injuries has been previously identified [7, 188–190]. Studies specifically aimed at preventing re-injuries were a minority, and were mostly related to recurrent ankle sprains.

Questions can be raised as to whether the identified ‘gaps’ in the number of studies evaluating the various intervention strategies represent actual knowledge gaps or are unavoidable as not all intervention strategies are appropriate for all sports, injury types and/or sport settings. This is illustrated by the differences found in intervention strategies used in studies of the prevention of soccer and rugby injuries. By its nature, rugby has a high injury rate due to the multiple contact situations [191]. This can explain the emphasis in rugby studies on intervention strategies related to PPE use and rules and regulations, as opposed to soccer. Similarly, differences between strategies used to prevent ankle and knee sprains, as opposed to concussions, can be related to the aetiology and mechanisms of these injuries [10, 192, 193]. However, all possible intervention strategies should be considered when first developing sport injury prevention programmes, and lessons can be learned from strategies used in other sports and injury types. As such, the Haddon matrix presented in this review is a useful tool to identify possible intervention strategies for sport injury prevention.

Based on this review, some knowledge gaps relating to effective sport injury prevention strategies can be identified. New research in these gap areas could be a valuable addition to the current knowledge base of sport injury prevention. This especially applies to research on rule modifications in sport as an intervention strategy. Most research in this area to date has focussed on the preventive effect of mandatory PPE use in the event phase. However, evidence on the effectiveness of rule modifications in the pre-event phase is scarce. Exceptions are two studies on the preventive effect of a new scrum law in rugby [154] and new karate rules [166]. Such strategies have the potential to limit or eliminate dangerous situations in play, and hence prevent sport injury events from occurring. Rule modifications can be of preventive value in the post-event phase as well, but no studies on this intervention strategy were found. To this category would belong rules that allow free substitution and off-field medical assessment during play to modify the risk of (recurrent) injuries [194]. Furthermore, although sport equipment has been a frequently studied topic in sport injury prevention, studies on the effect of equipment modifications in the pre-event phase are rare. Such preventive interventions do exist in real-world sport settings (e.g. different floor types, tyres to prevent falling in bicycle racing), but the potential preventive effect needs to be formally evaluated. Finally, only few studies were identified on the effect of training programmes other than those aimed at improving the physical skills of athletes. Additional studies are recommended to build on current evidence on the effect of improving psychological or cognitive skills, falling, landing and recovery skills, as well as education of athletes, coaches and referees. Overall, with the total number of 25 different sports considered in the studies included in this review, it is clear that many injury-prone sports have not yet been studied in the literature in this way (e.g. equestrian sport, tennis) [195].

The excess of RCTs used in sport injury prevention studies has been highlighted previously [11], and is not surprising as this study design is considered the gold standard for establishing the preventive effect of an intervention [14, 16, 196]. However, 43% of all injury prevention studies did not use a (randomised) controlled design. The Haddon approach showed that study design and intervention strategy are related. In studies evaluating strategies geared at rules and contextual modifications, RCTs/CTs were absent or a minority (17%). As most policies and rule modifications under study were introduced at a national level by a national sporting organisation or by law [25], randomisation was impossible and/or a proper control group was lacking. The effectiveness of these interventions could therefore not be evaluated using an RCT or CT design [14, 196]. The frequent use of pretest–posttest designs in these studies appears to be a justified option. Although alternative forms of RCTs have been suggested, including stepped wedge designs (in which an intervention at group level is sequentially implemented if randomisation is impossible) and Solomon four-group designs (to control for the effect of a pretest) [196, 197], these study designs have not yet been used in sport injury prevention studies to our knowledge. Consideration of the use of these designs may be of value in future sport injury prevention research to strengthen knowledge in this field, especially in studies evaluating the effect of group-based interventions.

Our review has some strengths and limitations. A systematic approach was used to identify all relevant sport injury prevention studies. Application of the pre-defined search strategy and inclusion and exclusion criteria resulted in the exclusion of studies not primarily targeting the evaluation of the efficacy or effectiveness of preventive interventions, for instance, aetiological studies establishing risk factors and injury mechanisms [7, 13]. Such studies may, however, provide valuable information related to specific intervention strategies, as illustrated by a study on the association between ice hockey injuries and arena characteristics [198]. The summary provided in this review identifies the amount of evidence (i.e. number of published studies and study designs used) and possible knowledge gaps per intervention strategy in a structured way using the modified Haddon matrix. This can support and strengthen future sport injury prevention efforts. However, additional information about the effectiveness, cost and feasibility of interventions is also necessary for practitioners in order to make a comprehensive decision on what strategy to use for sport injury prevention in everyday practice [199]. Neither did our review assess the effectiveness of preventive interventions, nor the risk of bias of individual studies (i.e. no assessment of the methodological quality of included studies) as per the purpose of this review. Also, an increasing number of implementation studies have been published in recent years [7], providing valuable information on effective implementation components in real-world sport settings [13, 18]. In this review, studies were also included that evaluated the effect of mandatory use of PPE and braces through rule modifications and policy changes. These intervention strategies represent a grey area between evaluating the preventive effect of an intervention and an implementation strategy. However, implementation of a new or modified rule should ideally be accompanied by implementation efforts at various levels [25].

In this review, we focussed on strategies used in the prevention of acute sport injuries, since the Haddon matrix was not developed for overuse injuries [26]. Only three studies exclusively targeting overuse injuries were excluded for this reason [32–34]. In addition, we limited our search to injury prevention studies reporting clinical outcomes, containing a quantitative injury measure as an outcome. As such, we excluded studies that reported on intermediate risk factors (e.g. biomechanical/physiological outcome measures) [200] and necessary behaviour changes related to sport injury risk as an outcome [201].

The current review may be subject to bias due to our literature search. We included five databases, and limited the search to English-language and peer-reviewed articles. Reference lists from recent systematic reviews and meta-analyses were manually searched for additional literature, which may have contributed to an overrepresentation of (randomised) controlled trials. Another possible source of bias was the exclusion of commuting activities (such as walking and cycling). As a result, studies of bicycle helmets in a general population were not included. These studies may have included helmet use in bicycle racing. However, no study was identified exclusively targeted at bicycle racing. The primary aim of each individual sport injury prevention study was used as a starting point for the categorisation of the extracted data. As a consequence, results of subgroup analyses that dealt with specific injury types or locations were not included in our categorisation.

## Conclusions

Using a modified version of the Haddon matrix, valuable insight into the extent of the evidence base of sport injury prevention studies was obtained for 20 potential intervention strategies, identifying the number of published studies and study designs used per strategy. This is a promising approach that could be used to monitor potential gaps in the knowledge base on sport injury prevention on an ongoing basis.

## References

[1] Warburton DE, Nicol CW, Bredin SS (2006). Health benefits of physical activity: the evidence. CMAJ.

[2] Blair SN (2007). Physical activity, clinical medicine, and public health. Curr Sports Med Rep.

[3] Haskell WL, Lee IM, Pate RR (2007). Physical activity and public health: updated recommendation for adults from the American College of Sports Medicine and the American Heart Association. Med Sci Sport Exerc.

[4] World Health Organization. Physical activity; fact sheet. 2016. http://www.who.int/mediacentre/factsheets/fs385/en/. Accessed 6 Nov 2016.

[5] Verhagen E, Bolling C, Finch CF (2015). Caution this drug may cause serious harm! Why we must report adverse effects of physical activity promotion. Br J Sports Med.

[6] Parkkari J, Kujala UM, Kannus P (2001). Is it possible to prevent sports injuries? Review of controlled clinical trials and recommendations for future work. Sports Med.

[7] Klügl M, Shrier I, McBain K (2010). The prevention of sport injury: an analysis of 12,000 published manuscripts. Clin J Sport Med.

[8] Aaltonen S, Karjalainen H, Heinonen A (2007). Prevention of sports injuries: systematic review of randomized controlled trials. Arch Intern Med.

[9] Leppänen M, Aaltonen S, Parkkari J (2014). Interventions to prevent sports related injuries: a systematic review and meta-analysis of randomised controlled trials. Sports Med.

[10] Steffen K, Andersen TE, Krosshaug T (2010). ECSS Position Statement 2009: prevention of acute sports injuries. Eur J Sport Sci.

[11] McBain K, Shrier I, Shultz R (2012). Prevention of sport injury II: a systematic review of clinical science research. Br J Sports Med.

[12] Lauersen JB, Bertelsen DM, Andersen LB (2014). The effectiveness of exercise interventions to prevent sports injuries: a systematic review and meta-analysis of randomised controlled trials. Br J Sports Med.

[13] Finch C (2006). A new framework for research leading to sports injury prevention. J Sci Med Sport.

[14] Emery C, Verhagen E, Van Mechelen W (2010). Research designs for evaluation studies. Sports injury research.

[15] Carey T, Sanders GD, Viswanathan M, et al. Appendix A, Taxonomy for study designs. Framework for considering study designs for future research needs (Methods Future Research Needs Reports, No 8). Rockville: Agency for Healthcare Research and Quality (US); 2012. 22624168

[16] Emery CA, Roos EM, Verhagen E (2015). OARSI clinical trials recommendations: design and conduct of clinical trials for primary prevention of osteoarthritis by joint injury prevention in sport and recreation. Osteoarthr Cartil.

[17] Verhagen E, Finch CF (2011). Setting our minds to implementation. Br J Sports Med.

[18] O’Brien J, Finch CF (2014). The implementation of musculoskeletal injury-prevention exercise programmes in team ball sports: a systematic review employing the RE-AIM framework. Sports Med.

[19] Finch CF (2011). No longer lost in translation: the art and science of sports injury prevention implementation research. Br J Sports Med.

[20] McGlashan AJ, Finch CF (2010). The extent to which behavioural and social sciences theories and models are used in sport injury prevention research. Sports Med.

[21] Verhagen E (2012). If athletes will not adopt preventive measures, effective measures must adopt athletes. Curr Sports Med Rep.

[22] Van Tiggelen D, Wickes S, Stevens V (2008). Effective prevention of sports injuries: a model integrating efficacy, efficiency, compliance and risk-taking behaviour. Br J Sports Med.

[23] Lund J, Aaro LE (2004). Accident prevention. Presentation of a model placing emphasis on human, structural and cultural factors. Saf Sci.

[24] Donaldson A, Verhagen E, Van Mechelen W (2010). The pragmatic approach. Sports injury research.

[25] Finch CF, Donaldson A (2010). A sports setting matrix for understanding the implementation context for community sport. Br J Sports Med.

[26] Haddon W (1980). Advances in the epidemiology of injuries as a basis for public policy. Public Health Rep.

[27] Bahr R, Karlsen R, Lian O (1994). Incidence and mechanisms of acute ankle inversion injuries in volleyball. A retrospective cohort study. Am J Sports Med.

[28] Hume PA, Lorimer AV, Griffiths PC (2015). Recreational snow-sports injury risk factors and countermeasures: a meta-analysis review and Haddon matrix evaluation. Sports Med.

[29] Fuller C, Verhagen E, Van Mechelen W (2010). Injury definitions. Sports injury research.

[30] Collard DC, Verhagen EA, Chinapaw MJ (2010). Effectiveness of a school-based physical activity injury prevention program: a cluster randomized controlled trial. Arch Pediatr Adolesc Med.

[31] Song JW, Chung KC (2010). Observational studies: cohort and case–control studies. Plast Reconstr Surg.

[32] Cobb KL, Bachrach LK, Sowers M (2007). The effect of oral contraceptives on bone mass and stress fractures in female runners. Med Sci Sport Exerc.

[33] Cumps E, Verhagen EA, Duerinck S (2008). Effect of a preventive intervention programme on the prevalence of anterior knee pain in volleyball players. Eur J Sport Sci.

[34] Hölmich P, Larsen K, Krogsgaard K (2010). Exercise program for prevention of groin pain in football players: a cluster-randomized trial. Scand J Med Sci Sports.

[35] Arnason A, Engebretsen L, Bahr R (2005). No effect of a video-based awareness program on the rate of soccer injuries. Am J Sports Med.

[36] Askling C, Karlsson J, Thorstensson A (2003). Hamstring injury occurrence in elite soccer players after preseason strength training with eccentric overload. Scand J Med Sci Sports.

[37] Barrett JR, Tanji JL, Drake C (1993). High- versus low-top shoes for the prevention of ankle sprains in basketball players. A prospective randomized study. Am J Sports Med.

[38] Bredeweg SW, Zijlstra S, Bessem B (2012). The effectiveness of a preconditioning programme on preventing running-related injuries in novice runners: a randomised controlled trial. Br J Sports Med.

[39] Buist I, Bredeweg SW, van Mechelen W (2008). No effect of a graded training program on the number of running-related injuries in novice runners: a randomized controlled trial. Am J Sports Med.

[40] Chaiwanichsiri D, Lorprayoon E, Noomanoch L (2005). Star excursion balance training: effects on ankle functional stability after ankle sprain. J Med Assoc Thai.

[41] Cusimano M, Luong WP, Faress A (2013). Evaluation of a ski and snowboard injury prevention program. Int J Inj Contr Saf Promot.

[42] Eils E, Schröter R, Schröder M (2010). Multistation proprioceptive exercise program prevents ankle injuries in basketball. Med Sci Sport Exerc.

[43] Ekstrand J, Gillquist J, Liljedahl SO (1983). Prevention of soccer injuries. Supervision by doctor and physiotherapist. Am J Sports Med.

[44] Emery CA, Cassidy JD, Klassen TP (2005). Effectiveness of a home-based balance-training program in reducing sports-related injuries among healthy adolescents: a cluster randomized controlled trial. CMAJ.

[45] Emery CA, Meeuwisse WH (2010). The effectiveness of a neuromuscular prevention strategy to reduce injuries in youth soccer: a cluster-randomised controlled trial. Br J Sports Med.

[46] Emery CA, Rose MS, McAllister JR (2007). A prevention strategy to reduce the incidence of injury in high school basketball: a cluster randomized controlled trial. Clin J Sport Med.

[47] Engebretsen AH, Myklebust G, Holme I (2008). Prevention of injuries among male soccer players—a prospective, randomized intervention study targeting players with previous injuries or reduced function. Am J Sports Med.

[48] Finch C, Braham R, McIntosh A (2005). Should football players wear custom fitted mouthguards? Results from a group randomised controlled trial. Inj Prev.

[49] Fredberg U, Bolvig L, Andersen NT (2008). Prophylactic training in asymptomatic soccer players with ultrasonographic abnormalities in Achilles and patellar tendons: the Danish Super League Study. Am J Sports Med.

[50] Garrick JG, Requa RK (1973). Role of external support in the prevention of ankle sprains. Med Sci Sports.

[51] Gilchrist J, Mandelbaum BR, Melancon H (2008). A randomized controlled trial to prevent noncontact anterior cruciate ligament injury in female collegiate soccer players. Am J Sports Med.

[52] Hägglund M, Walden M, Ekstrand J (2007). Lower reinjury rate with a coach-controlled rehabilitation program in amateur male soccer: a randomized controlled trial. Am J Sports Med.

[53] Hammes D, Fuenten KAD, Kaiser S (2015). Injury prevention in male veteran football players—a randomised controlled trial using “FIFA 11+”. J Sport Sci.

[54] Heidt RS, Sweeterman LM, Carlonas RL (2000). Avoidance of soccer injuries with preseason conditioning. Am J Sports Med.

[55] Hides J, Stanton W (2014). Can motor control training lower the risk of injury for professional football players?. Med Sci Sport Exerc.

[56] Holme E, Magnusson SP, Becher K (1999). The effect of supervised rehabilitation on strength, postural sway, position sense and re-injury risk after acute ankle ligament sprain. Scand J Med Sci Sports.

[57] Barbic D, Pater J, Brison RJ (2005). Comparison of mouth guard designs and concussion prevention in contact sports: a multicenter randomized controlled trial. Clin J Sport Med.

[58] Frey C, Feder KS, Sleight J (2010). Prophylactic ankle brace use in high school volleyball players: a prospective study. Foot Ankle Int.

[59] Hupperets MD, Verhagen EA, van Mechelen W (2009). Effect of unsupervised home based proprioceptive training on recurrences of ankle sprain: randomised controlled trial. BMJ.

[60] Jamtvedt G, Herbert RD, Flottorp S (2010). A pragmatic randomised trial of stretching before and after physical activity to prevent injury and soreness. Br J Sports Med.

[61] Jorgensen U, Fredensborg T, Haraszuk JP (1998). Reduction of injuries in downhill skiing by use of an instructional ski-video: a prospective randomised intervention study. Knee Surg Sports Traumatol Arthrosc.

[62] Kinchington MA, Ball KA, Naughton G (2011). Effects of footwear on comfort and injury in professional rugby league. J Sport Sci.

[63] LaBella CR, Huxford MR, Grissom J (2011). Effect of neuromuscular warm-up on injuries in female soccer and basketball athletes in urban public high schools: cluster randomized controlled trial. Arch Pediatr Adolesc Med.

[64] Longo UG, Loppini M, Berton A (2012). The FIFA 11+ program is effective in preventing injuries in elite male basketball players: a cluster randomized controlled trial. Am J Sports Med.

[65] Machold W, Kwasny O, Eisenhardt P (2002). Reduction of severe wrist injuries in snowboarding by an optimized wrist protection device: a prospective randomized trial. J Trauma.

[66] McGuine TA, Keene JS (2006). The effect of a balance training program on the risk of ankle sprains in high school athletes. Am J Sports Med.

[67] McGuine TA, Hetzel S, Wilson J (2012). The effect of lace-up ankle braces on injury rates in high school football players. Am J Sports Med.

[68] McIntosh AS, McCrory P, Finch CF (2009). Does padded headgear prevent head injury in rugby union football?. Med Sci Sport Exerc.

[69] Mohammadi F (2007). Comparison of 3 preventive methods to reduce the recurrence of ankle inversion sprains in male soccer players. Am J Sports Med.

[70] Petersen J, Thorborg K, Nielsen MB (2011). Preventive effect of eccentric training on acute hamstring injuries in men’s soccer: a cluster-randomized controlled trial. Am J Sports Med.

[71] Pasanen K, Parkkari J, Pasanen M (2008). Neuromuscular training and the risk of leg injuries in female floorball players: cluster randomised controlled study. BMJ.

[72] Owoeye OB, Akinbo SR, Tella BA (2014). Efficacy of the FIFA 11+ warm-up programme in male youth football: a cluster randomised controlled trial. J Sport Sci Med..

[73] Olsen OE, Myklebust G, Engebretsen L (2005). Exercises to prevent lower limb injuries in youth sports: cluster randomised controlled trial. BMJ.

[74] Sitler M, Ryan J, Hopkinson W (1990). The efficacy of prophylactic knee brace to reduce knee injuries in football—a prospective, randomized study at West Point. Am J Sports Med.

[75] Ronning R, Ronning I, Gerner T (2001). The efficacy of wrist protectors in preventing snowboarding injuries. Am J Sports Med.

[76] van Beijsterveldt AM, van de Port IG, Krist MR (2012). Effectiveness of an injury prevention programme for adult male amateur soccer players: a cluster-randomised controlled trial. Br J Sports Med.

[77] Tropp H, Askling C, Gillquist J (1985). Prevention of ankle sprains. Am J Sports Med.

[78] Walden M, Atroshi I, Magnusson H (2012). Prevention of acute knee injuries in adolescent female football players: cluster randomised controlled trial. BMJ.

[79] Verhagen E, van der Beek A, Twisk J (2004). The effect of a proprioceptive balance board training program for the prevention of ankle sprains: a prospective controlled trial. Am J Sports Med.

[80] van Mechelen W, Hlobil H, Kemper HC (1993). Prevention of running injuries by warm-up, cool-down, and stretching exercises. Am J Sports Med.

[81] Steffen K, Myklebust G, Olsen OE (2008). Preventing injuries in female youth football—a cluster-randomized controlled trial. Scand J Med Sci Sports.

[82] Surve I, Schwellnus MP, Noakes T (1994). A fivefold reduction in the incidence of recurrent ankle sprains in soccer players using the Sport-Stirrup orthosis. Am J Sports Med.

[83] Winters J, DeMont R (2014). Role of mouthguards in reducing mild traumatic brain injury/concussion incidence in high school football athletes. Gen Dent.

[84] Wedderkopp N, Kaltoft M, Holm R (2003). Comparison of two intervention programmes in young female players in European handball—with and without ankle disc. Scand J Med Sci Sports.

[85] Wedderkopp N, Kaltoft M, Lundgaard B (1999). Prevention of injuries in young female players in European team handball. A prospective intervention study. Scand J Med Sci Sports.

[86] Kolt GS, Hume PA, Smith P (2004). Effects of a stress-management program on injury and stress of competitive gymnasts. Percept Mot Skills.

[87] Kraus JF, Anderson BD, Mueller CE (1970). An investigation of the effectiveness of a new helmet to control touch football head injuries. Am J Public Health Nations Health.

[88] Sebelien C, Stiller C, Maher S (2014). Effects of implementing Nordic hamstring exercises for semi-professional soccer players in Akershus, Norway. Orthop Pract.

[89] Söderman K, Werner S, Pietila T (2000). Balance board training: prevention of traumatic injuries of the lower extremities in female soccer players? A prospective randomized intervention study. Knee Surg Sports Traumatol Arthrosc.

[90] Soligard T, Myklebust G, Steffen K (2008). Comprehensive warm-up programme to prevent injuries in young female footballers: cluster randomised controlled trial. BMJ.

[91] Silvers-Granelli H, Mandelbaum B, Adeniji O (2015). Efficacy of the FIFA 11+ Injury Prevention Program in the collegiate male soccer player. Am J Sports Med.

[92] Aerts I, Cumps E, Verhagen E (2013). A 3-month jump-landing training program: a feasibility study using the RE-AIM framework. J Athl Train.

[93] Johnson U, Ekengren J, Andersen MB (2005). Injury prevention in Sweden: helping soccer players at risk. J Sport Exerc Psychol.

[94] Maddison R, Prapavessis H (2005). A psychological approach to the prediction and prevention of athletic injury. J Sport Exerc Psychol.

[95] Sitler M, Ryan J, Wheeler B (1994). The efficacy of a semirigid ankle stabilizer to reduce acute ankle injuries in basketball. A randomized clinical study at West Point. Am J Sports Med.

[96] Kraus JF, Anderson BD, Mueller CE (1971). The quality of officiating as an injury prevention factor in intramural touch football. Med Sci Sports..

[97] McGuine TA, Brooks A, Hetzel S (2011). The effect of lace-up ankle braces on injury rates in high school basketball players. Am J Sports Med.

[98] Zakaria AA, Kiningham RB, Sen A (2015). Effects of static and dynamic stretching on injury prevention in high school soccer athletes: a randomized trial. J Sport Rehabil.

[99] Wester JU, Jespersen SM, Nielsen KD (1996). Wobble board training after partial sprains of the lateral ligaments of the ankle: a prospective randomized study. J Orthop Sport Phys.

[100] Bixler B, Jones RL (1992). High-school football injuries: effects of a post-halftime warm-up and stretching routine. Fam Pract Res J.

[101] Caraffa A, Cerulli G, Projetti M (1996). Prevention of anterior cruciate ligament injuries in soccer. A prospective controlled study of proprioceptive training. Knee Surg Sports Traumatol Arthrosc.

[102] Croisier JL, Ganteaume S, Binet J (2008). Strength imbalances and prevention of hamstring injury in professional soccer players: a prospective study. Am J Sports Med.

[103] Cumps E, Verhagen E, Meeusen R (2007). Efficacy of a sports specific balance training programme on the incidence of ankle sprains in basketball. J Sport Sci Med.

[104] Danis RP, Hu K, Bell M (2000). Acceptability of baseball face guards and reduction of oculofacial injury in receptive youth league players. Inj Prev.

[105] de Hoyo M, Pozzo M, Sanudo B (2015). Effects of a 10-week in-season eccentric-overload training program on muscle-injury prevention and performance in junior elite soccer players. Int J Sports Physiol Perform.

[106] Edvardsson A, Ivarsson A, Johnson U (2012). Is a cognitive-behavioural biofeedback intervention useful to reduce injury risk in junior football players?. J Sport Sci Med.

[107] Gatterer H, Ruedl G, Faulhaber M (2012). Effects of the performance level and the FIFA “11” injury prevention program on the injury rate in Italian male amateur soccer players. J Sport Med Phys Fit.

[108] Jakobsen BW, Kroner K, Schmidt SA (1994). Prevention of injuries in long-distance runners. Knee Surg Sports Traumatol Arthrosc.

[109] Janda DH, Wojtys EM, Hankin FM (1988). Softball sliding injuries. A prospective study comparing standard and modified bases. JAMA.

[110] Junge A, Rosch D, Peterson L (2002). Prevention of soccer injuries: a prospective intervention study in youth amateur players. Am J Sports Med.

[111] Kiani A, Hellquist E, Ahlqvist K (2010). Prevention of soccer-related knee injuries in teenaged girls. Arch Intern Med.

[112] Malliou P, Gioftsidou A, Pafis G (2004). Proprioceptive training (balance exercises) reduces lower extremity injuries in young soccer players. J Back Musculoskelet.

[113] Mandelbaum BR, Silvers HJ, Watanabe DS (2005). Effectiveness of a neuromuscular and proprioceptive training program in preventing anterior cruciate ligament injuries in female athletes: 2-year follow-up. Am J Sports Med.

[114] Mitchell B (2000). Efficacy of thigh protectors in preventing thigh haematomas. J Sci Med Sport.

[115] Moiler K, Hall T, Robinson K (2006). The role of fibular tape in the prevention of ankle injury in basketball: a pilot study. J Orthop Sport Phys.

[116] Petersen W, Braun C, Bock W (2005). A controlled prospective case control study of a prevention training program in female team handball players: the German experience. Arch Orthop Trauma Surg.

[117] Pfeiffer RP, Shea KG, Roberts D (2006). Lack of effect of a knee ligament injury prevention program on the incidence of noncontact anterior cruciate ligament injury. J Bone Joint Surg Am.

[118] Scase E, Cook J, Makdissi M (2006). Teaching landing skills in elite junior Australian football: evaluation of an injury prevention strategy. Br J Sports Med.

[119] Timpka T, Lindqvist K (2001). Evidence based prevention of acute injuries during physical exercise in a WHO safe community. Br J Sports Med.

[120] Moon DG, Mitchell DF (1961). An evaluation of a commercial protective mouthpiece for football players. J Am Dent Assoc.

[121] Kerr G, Goss J (1996). The effects of a stress management program on injuries and stress levels. J Appl Sport Psychol.

[122] Tranaeus U, Johnson U, Engstrom B (2015). A psychological injury prevention group intervention in Swedish floorball. Knee Surg Sports Traumatol Arthrosc.

[123] Albright JP, Powell JW, Smith W (1994). Medial collateral ligament knee sprains in college football. Effectiveness of preventive braces. Am J Sports Med.

[124] Al-Habib A, Attabib N, Hurlbert RJ (2012). Recreational helmet use as a predictor of noncranial injury. J Trauma.

[125] Benson BW, Mohtadi NG, Rose MS (1999). Head and neck injuries among ice hockey players wearing full face shields vs half face shields. JAMA.

[126] Brooks JH, Fuller CW, Kemp SP (2006). Incidence, risk, and prevention of hamstring muscle injuries in professional rugby union. Am J Sports Med.

[127] Deppen RJ, Landfried MJ (1994). Efficacy of prophylactic knee bracing in high school football players. J Orthop Sport Phys.

[128] Hejna WF, Rosenberg A, Buturusis DJ (1982). Prevention of sports injuries in high school students through strength training. NSCA J.

[129] Hewett TE, Lindenfeld TN, Riccobene JV (1999). The effect of neuromuscular training on the incidence of knee injury in female athletes. A prospective study. Am J Sports Med.

[130] Brunelle JP, Goulet C, Arguin H (2005). Promoting respect for the rules and injury prevention in ice hockey: evaluation of the fair-play program. J Sci Med Sport.

[131] Curtis CK, Laudner KG, McLoda TA (2008). The role of shoe design in ankle sprain rates among collegiate basketball players. J Athl Train.

[132] Grace TG, Skipper BJ, Newberry JC (1988). Prophylactic knee braces and injury to the lower extremity. J Bone Jt Surg Am.

[133] Johannsen HV, Noerregaard FO (1988). Prevention of injury in karate. Br J Sports Med.

[134] Kriz PK, Comstock RD, Zurakowski D (2012). Effectiveness of protective eyewear in reducing eye injuries among high school field hockey players. Pediatrics.

[135] Macpherson A, Rothman L, Howard A (2006). Body-checking rules and childhood injuries in ice hockey. Pediatrics.

[136] Marshall SW, Mueller FO, Kirby DP (2003). Evaluation of safety balls and faceguards for prevention of injuries in youth baseball. JAMA.

[137] Marshall SW, Loomis DP, Waller AE (2005). Evaluation of protective equipment for prevention of injuries in rugby union. Int J Epidemiol.

[138] Pedowitz DI, Reddy S, Parekh SG (2008). Prophylactic bracing decreases ankle injuries in collegiate female volleyball players. Am J Sports Med.

[139] Teitz CC, Hermanson BK, Kronmal RA (1987). Evaluation of the use of braces to prevent injury to the knee in collegiate football players. J Bone Jt Surg Am.

[140] Upton PAH, Noakes TD, Juritz JM (1996). Thermal pants may reduce the risk of recurrent hamstring injuries in rugby players. Br J Sports Med.

[141] Webster DA, Bayliss GV, Spadaro JA (1999). Head and face injuries in scholastic women’s lacrosse with and without eyewear. Med Sci Sport Exerc.

[142] Yang J, Marshall SW, Bowling JM (2005). Use of discretionary protective equipment and rate of lower extremity injury in high school athletes. Am J Epidemiol.

[143] McIntosh AS, McCrory P (2001). Effectiveness of headgear in a pilot study of under 15 rugby union football. Br J Sports Med.

[144] Seagrave RA, Perez L, McQueeney S (2014). Preventive effects of eccentric training on acute hamstring muscle injury in professional baseball. Orthop J Sports Med.

[145] Abu-Zidan FM, Hefny AF, Branicki F (2012). Prevention of child camel jockey injuries: a success story from the United Arab Emirates. Clin J Sport Med.

[146] Arnason A, Andersen TE, Holme I (2008). Prevention of hamstring strains in elite soccer: an intervention study. Scand J Med Sci Sports.

[147] Bahr R, Lian O, Bahr IA (1997). A twofold reduction in the incidence of acute ankle sprains in volleyball after the introduction of an injury prevention program: a prospective cohort study. Scand J Med Sci Sports.

[148] Bjorneboe J, Bahr R, Dvorak J (2013). Lower incidence of arm-to-head contact incidents with stricter interpretation of the Laws of the Game in Norwegian male professional football. Br J Sports Med.

[149] Brown JC, Verhagen E, Knol D (2016). The effectiveness of the nationwide BokSmart rugby injury prevention program on catastrophic injury rates. Scand J Med Sci Sports.

[150] Burtscher M, Gatterer H, Flatz M (2008). Effects of modern ski equipment on the overall injury rate and the pattern of injury location in alpine skiing. Clin J Sport Med.

[151] Dvorak J, Junge A, Grimm K (2007). Medical report from the 2006 FIFA World Cup Germany. Br J Sports Med.

[152] Elena-Doina M, Mogaseanu M, Dunarintu S (2011). Prevention of musculo-skeletal traumas in competitive sportsmen: (aspects regarding trauma incidence in volleyball and basketball teams). Ovidius Univ Ann Ser Phys Educ Sport/Sci Mov Health.

[153] Elphinston J, Hardman SL (2006). Effect of an integrated functional stability program on injury rates in an international netball squad. J Sci Med Sport.

[154] Gianotti S, Hume PA, Hopkins WG (2008). Interim evaluation of the effect of a new scrum law on neck and back injuries in rugby union. Br J Sports Med.

[155] Gianotti SM, Quarrie KL, Hume PA (2009). Evaluation of RugbySmart: a rugby union community injury prevention programme. J Sci Med Sport.

[156] Grooms DR, Palmer T, Onate JA (2013). Soccer-specific warm-up and lower extremity injury rates in collegiate male soccer players. J Athl Train.

[157] Hadala M, Barrios C (2009). Different strategies for sports injury prevention in an America’s Cup yachting crew. Med Sci Sport Exerc.

[158] Hagel BE, Marko J, Dryden D (2006). Effect of bodychecking on injury rates among minor ice hockey players. CMAJ.

[159] Harris AW, Voaklander DC, Drul C (2012). Hockey-related emergency department visits after a change in minor hockey age groups. Clin J Sport Med.

[160] Bollars P, Claes S, Vanlommel L (2014). The effectiveness of preventive programs in decreasing the risk of soccer injuries in Belgium: national trends over a decade. Am J Sports Med.

[161] Cusimano MD, Taback NA, McFaull SR (2011). Effect of bodychecking on rate of injuries among minor hockey players. Open Med.

[162] Goossens L, Cardon G, Witvrouw E (2016). A multifactorial injury prevention intervention reduces injury incidence in Physical Education Teacher Education students. Eur J Sport Sci.

[163] Junge A, Lamprecht M, Stamm H (2011). Countrywide campaign to prevent soccer injuries in Swiss amateur players. Am J Sports Med.

[164] Kaplan Y, Myklebust G, Nyska M (2014). The prevention of injuries in contact flag football. Knee Surg Sports Traumatol Arthrosc.

[165] Lehnhard RA, Lehnhard HR, Young R (1996). Monitoring injuries on a college soccer team: the effect of strength training. J Strength Cond Res.

[166] Macan J, Bundalo-Vrbanac D, Romic G (2006). Effects of the new karate rules on the incidence and distribution of injuries. Br J Sports Med.

[167] McHugh MP, Tyler TF, Mirabella MR (2007). The effectiveness of a balance training intervention in reducing the incidence of noncontact ankle sprains in high school football players. Am J Sports Med.

[168] Myklebust G, Engebretsen L, Braekken IH (2003). Prevention of anterior cruciate ligament injuries in female team handball players: a prospective intervention study over three seasons. Clin J Sport Med.

[169] Quarrie KL, Gianotti SM, Chalmers DJ (2005). An evaluation of mouthguard requirements and dental injuries in New Zealand rugby union. Br J Sports Med.

[170] Owen AL, del Wong P, Dellal A (2013). Effect of an injury prevention program on muscle injuries in elite professional soccer. J Strength Cond Res..

[171] Rovere GD, Haupt HA, Yates CS (1987). Prophylactic knee bracing in college football. Am J Sports Med.

[172] Shaw L, Finch CF (2008). Injuries to junior club cricketers: the effect of helmet regulations. Br J Sports Med.

[173] Verrall GM, Slavotinek JP, Barnes PG (2005). The effect of sports specific training on reducing the incidence of hamstring injuries in professional Australian Rules football players. Br J Sports Med.

[174] Tyler TF, Nicholas SJ, Campbell RJ (2002). The effectiveness of a preseason exercise program to prevent adductor muscle strains in professional ice hockey players. Am J Sports Med.

[175] Ytterstad B (1996). The Harstad injury prevention study: the epidemiology of sports injuries. An 8 year study. Br J Sports Med.

[176] Kraemer R, Knobloch K (2009). A soccer-specific balance training program for hamstring muscle and patellar and Achilles tendon injuries: an intervention study in premier league female soccer. Am J Sports Med.

[177] Kraus JF, Anderson BD, Mueller CE (1970). The effectiveness of a special ice hockey helmet to reduce head injuries in college intramural hockey. Med Sci Sports.

[178] Slaney GM, Weinstein P (2009). Community-driven intervention to reduce injury rates in school-age snowboarders. Aust J Rural Health.

[179] Cahill BR, Griffith EH (1978). Effect of preseason conditioning on the incidence and severity of high school football knee injuries. Am J Sports Med.

[180] Ettlinger CF, Johnson RJ, Shealy JE (1995). A method to help reduce the risk of serious knee sprains incurred in alpine skiing. Am J Sports Med.

[181] Melegati G, Tornese D, Gevi M (2013). Reducing muscle injuries and reinjuries in one Italian professional male soccer team. Muscles Ligaments Tendons J.

[182] Gianotti S, Hume PA (2007). Concussion sideline management intervention for rugby union leads to reduced concussion claims. Neurorehabilitation.

[183] Kriz PK, Zurakowski D, Almquist JL (2015). Eye protection and risk of eye injuries in high school field hockey. Pediatrics.

[184] Lincoln AE, Caswell SV, Almquist JL (2012). Effectiveness of the women’s lacrosse protective eyewear mandate in the reduction of eye injuries. Am J Sports Med.

[185] Kukaswadia A, Warsh J, Mihalik JP (2010). Effects of changing body-checking rules on rates of injury in minor hockey. Pediatrics.

[186] Quarrie KL, Gianotti SM, Hopkins WG (2007). Effect of nationwide injury prevention programme on serious spinal injuries in New Zealand rugby union: ecological study. BMJ.

[187] Vriend I, Valkenberg H, Schoots W (2015). Shinguards effective in preventing lower leg injuries in football: population-based trend analyses over 25 years. J Sci Med Sport.

[188] Orchard JW, McCrory P, Makdissi M (2014). Use of rule changes to reduce injury in the Australian Football League. Minerva Ortop Trauma.

[189] Arias JL, Argudo FM, Alonso JI (2011). Review of rule modification in sport. J Sport Sci Med.

[190] Matheson GO, Mohtadi NG, Safran M (2010). Sport injury prevention: time for an intervention?. Clin J Sport Med.

[191] Fuller CW, Brooks JH, Cancea RJ (2007). Contact events in rugby union and their propensity to cause injury. Br J Sports Med.

[192] Khurana VG, Kaye AH (2012). An overview of concussion in sport. J Clin Neurosci.

[193] Verhagen E (2013). How fundamental knowledge aids implementation: ankle sprains as an example. Acta Medica Port.

[194] McCrory P, Meeuwisse W, Johnston K (2009). Consensus statement on concussion in sport—the 3rd international conference on concussion in sport, held in Zurich, November 2008. J Clin Neurosci.

[195] Schmikli SL, Backx FJ, Kemler HJ (2009). National survey on sports injuries in the Netherlands: target populations for sports injury prevention programs. Clin J Sport Med.

[196] Carey T, Sanders GD, Viswanathan M, et al. Appendix A, taxonomy for study designs. Framework for considering study designs for future research needs (methods future research needs reports, No 8). 2012. http://www.ncbi.nlm.nih.gov/books/NBK95280/. Accessed 25 March 2016.

[197] Beard E, Lewis JJ, Copas A (2015). Stepped wedge randomised controlled trials: systematic review of studies published between 2010 and 2014. Trials.

[198] Tuominen M, Stuart MJ, Aubry M (2015). Injuries in men’s international ice hockey: a 7-year study of the International Ice Hockey Federation Adult World Championship Tournaments and Olympic Winter Games. Br J Sports Med.

[199] Runyan CW (2015). Using the Haddon matrix: introducing the third dimension. Inj Prev.

[200] McBain K, Shrier I, Shultz R (2012). Prevention of sports injury I: a systematic review of applied biomechanics and physiology outcomes research. Br J Sports Med.

[201] Verhagen EA, van Stralen MM, van Mechelen W (2010). Behaviour, the key factor for sports injury prevention. Sports Med.

